# Population-Based Evidence of Climate Change Adaptation in an Endangered Plant Endemic to a Biodiversity Hotspot

**DOI:** 10.3390/plants12102017

**Published:** 2023-05-18

**Authors:** Diego Alarcón, David Santos, Mary T. K. Arroyo

**Affiliations:** 1 Departamento de Ciencias Ecológicas, Universidad de Chile, Las Palmeras 3425, Ñuñoa, Santiago 7800003, Chile; southern@uchile.cl; 2 Instituto de Ecología y Biodiversidad (IEB), Las Palmeras 3425, Ñuñoa, Santiago 7800003, Chile; 3 Vivero Encanto Salvaje, Callejón San Martín 22, Linares 3580000, Chile; davidsantosbecerra@gmail.com; 4 Cape Horn International Center (CHIC), O’Higgins 310, Cabo de Hornos 6350001, Chile

**Keywords:** climate change, vulnerability, adaptation, species distribution models, threatened species, population structure, *Anemone*, *Knowltonia*, Chile

## Abstract

Climate change is expected to impact both the population structure and geographic distribution of plants. Species distribution models are widely used to assess range shifts and the vulnerability of plants to climate change. Despite the abundance of modeling studies, little is known about how existing populations respond to climate change. We investigated the demographic structure and vulnerability to climate change in *Anemone moorei*, a sub-shrub with a highly restricted distribution in a biodiversity hotspot. We improved the distribution knowledge through intensive field work. We conducted a census of stem length as a proxy for age for all known populations. We used ensemble forecasting to project distributions considering 10 future climate scenarios and developed a novel climate change vulnerability index for the species’ distribution. We found that the mean stem length decreases and the proportion of young plants increases, while the size of fruiting plants decreases as *A. moorei* faces greater climate change vulnerability. We interpret these results as evidence for the onset of recent adaptation to climate change, consisting of reduced adult longevity and an earlier onset of reproduction. As a result of these changes, the proportion of juveniles in the population increases.

## 1. Introduction

Climate change is expected to have long-term negative impacts on biodiversity [1,2,3,4], leading to changes in developmental responses [1,5,6,7,8,9,10], phenology [11,12,13,14], plasticity [15,16], genetic structure [17,18,19,20], growth rates [21,22], regeneration [23,24], and shifts in species’ distributions [25,26,27,28,29]. Among the various methods used to assess the effect of climate change, species distribution models (SDMs), based on the ecological niche theory, are among the most widely used [30,31]. Such models relate the location of species to the ranges of environmental variables under which they can live, grow, and reproduce in a given period of time, and predict distributions in geographic space or time according to future climate models [32,33,34,35]. Many studies that have used SDMs to predict changes in the geographic range of species populations under climate change show expansion into new areas where the conditions are suitable and species can migrate, as well as reductions in areas that are projected to be outside their suitable ecological ranges [36,37,38,39,40,41]. Populations of a species can be classified according to their vulnerability to climate change by comparing current and future projections using SDMs. Thus, populations where environmental suitability becomes less adequate will be more vulnerable to climate change [36,37,42].

Despite the abundance of studies modeling future projections with SDMs, there is little information on the demographic parameters of the existing populations that are projected to experience such changes [43]. If the population structure responds rapidly, local adaptation, rather than wholesale migration, could be expected [18,44].

Although SDMs are not a tool for modeling fine demographic parameters, they have proven to be successful for predicting the occurrence of plant species [6,45,46]. Therefore, for a population that is predicted by an SDM to become unsuitable, early evidence of changes in regeneration or adult plant longevity that are consistent with the direction of the prediction is to be expected [47,48,49].

Endemic species with restricted distributions are considered more vulnerable to the threat of climate change compared to more widely distributed species with broader ecological niches [36,50]. A very restricted distribution is also one of the criteria used by the International Union for Conservation of Nature (IUCN) to classify a species as threatened [51] and is therefore expected to have a greater impact in the face of climate change.

Biodiversity hotspots are high-priority ecosystems for conservation because they contain a significant proportion of endemic species, are subject to high-impact threats [52,53], and are highly vulnerable to climate change [54]. The South American Andes contain important biodiversity hotspots and are considered a natural laboratory for studying the effects of climate change on plant biodiversity [41,55]. The “Chilean Winter Rainfall-Valdivian Forests” biodiversity hotspot is important because it encompasses a significant portion of the southern Andes with high species endemism, especially of plants [56]. 

Increasing temperature and decreasing precipitation are projected for the Chilean Winter Rainfall-Valdivian Forests biodiversity hotspot [57], with rapid ongoing changes in these climatic factors along the entire hotspot already underway [58]. Using SDMs, climate data have been used to project changes in the distribution of different plant species in this hotspot [59,60,61] and in plant growth rate [21]. This scenario provides an opportunity to examine possible population-level adjustments that are consistent with the changes predicted by SDMs under climate change scenarios.

We hypothesize that for a plant species with a narrow ecological niche endemic to a biodiversity hotspot, populations predicted by an SDM to be more vulnerable to climate change should show greater demographic impacts compared to populations predicted to be less vulnerable to climate change. The main objective of our work was to assess whether the differences in the demographic structure of populations of a plant endemic to a biodiversity hotspot reflect the differences in the vulnerability of its populations to climate change, as predicted by an SDM. 

## 2. Materials and Methods

### 2.1. Species Selection

We searched for a species that met the following requirements: (i) Species for which it was possible to determine the demographic structure based on a trait that was measurable in the field. (ii) Species characterized by a uniform habitat and not subject to intensive exploitation so as to avoid non-climate-related impacts on demographic structure. (iii) Species with a restricted distribution associated with a narrow ecological niche and classified as “endangered” or “critically endangered” according to IUCN [51,62], as applied by the Chilean species classification process [63].

**Figure 1:**
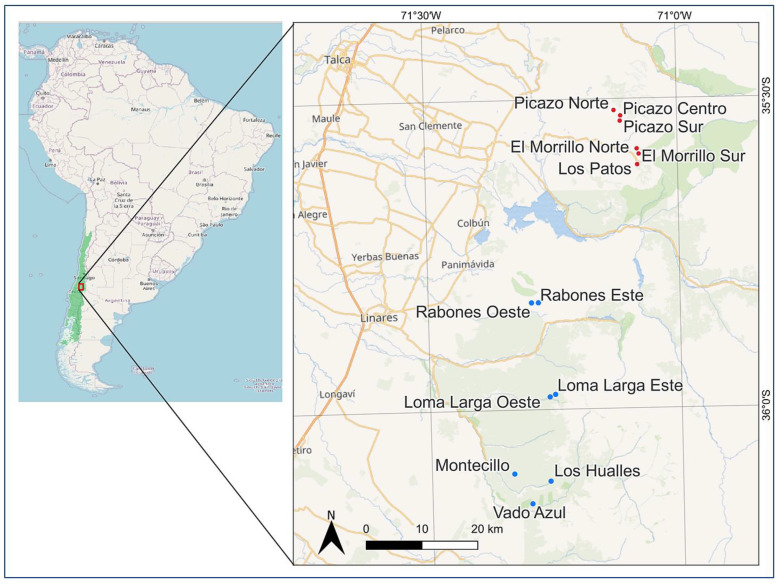
Location of the study area and populations. In the South American map on the left, the green shading represents the “Chilean Winter Rainfall-Valdivian Forests” biodiversity hotspot. In the red square zoomed in on the right, red dots indicate previously known *A. moorei* populations, while blue dots indicate newly reported populations during this study.

**Figure 2:**
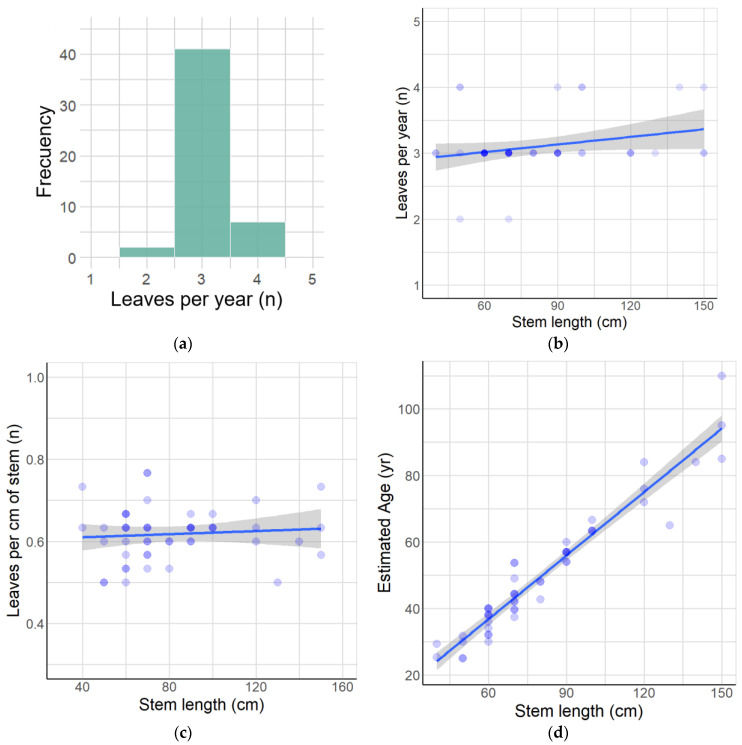
Growth characteristics of *A. moorei.* (**a**) Frequency of new leaves per year. (**b**) Relationship between leaves per year and stem length. (**c**) Relationship between leaves per cm of stem and stem length. (**d**) Estimated plant age according to stem length.

One species that meets the above criteria is *Anemone moorei* Espinosa (=*Knowltonia moorei* (Espinosa) Christenh. & Byng), Ranunculaceae (Figure S1), a sub-shrub and a nanophanerophyte according to Raunkiaer’s classification [64], which is endemic to the foothills of the Andes in the Maule region of Chile [65,66,67,68,69]. *A. moorei* is currently known from thirteen populations (Figure 1). With respect to requirement (i), our preliminary observations showed that stems of *A. moorei* produce ca. 3 leaves per year (Figure 2a,b, data in Table S1). In addition, the stem increment associated with the annual addition of three leaves is similar for plants of all ages (Figure 2c and Figure S2). This means that stem length, which is easily measured in the field, can be used as a comparative proxy for individual plant age in this particular species (see Figure 2d, data in Table S1). With regard to requirement (ii), *A. moorei* is restricted to the understory of non-disturbed forests dominated by two deciduous species of *Nothofagus* (*N. obliqua* and *N. alpina*), which form a uniform canopy. Consequently, populations on all sites are subject to similar local environmental conditions. In relation to requirement (iii), up to the time of the present study, *A. moorei* was known from only six populations. It is classified as an endangered species (EN B1ab(iii) + 2ab(iii)) due to its very restricted distribution and limited number of locations [70]. 

### 2.2. Census and Population Characterization by Site 

*A. moorei* was only known from the following six populations prior to this study: Picazo Norte, Picazo Centro, Picazo Sur, El Morrillo Norte, El Morrillo Sur, and Los Patos. The known geographical range of *A. moorei* and adjacent areas with similar environmental characteristics where the species was not previously known to occur were intensively searched. Our extensive survey revealed a total of seven new populations (Figure 1), all to the south of the previously known populations. The new populations are as follows: Rabones Oeste, Rabones Este, Loma Larga Este, Loma Larga Oeste, Montecillo, Los Hualles, and Vado Azul. 

All known individuals in all populations of the species were georeferenced with a GarminTM 62s GPS. We recorded stem length for each plant, classifying them as reproductive (flowering plant and fruiting plant) and non-reproductive. When an individual plant had more than one stem, the length of the longest stem was recorded.

The demographic structure of the *A. moorei* population at each site was characterized by histograms showing the distribution of stem length frequencies and the skewness of the respective distributions. At each site, mean stem length and other parameters such as the proportion of young and adult plants were determined. Plants were classified as young plants using a stem length threshold of less than or equal to 30 cm. Less than 5% of the plants of this size were able to produce flowers. Plants with stem lengths above 30 cm were considered adult plants. 

In order to determine the size of the area in which the species develops at each site, the geographic coordinates of *A. moorei* plants were evaluated with the convexhull function of the R package spatialEco [71] using the alphahull method [72]. This allowed us to obtain the adjusted polygon bounding individuals of the species at each site. QGIS software [73] was used to calculate the area of each polygon and the mean distance between plants at each site. The area occupied by each population was used to calculate the density of young and adult individuals.

### 2.3. Species Distribution Models for Current and Future Scenarios

The current distribution of *A. moorei* was modeled using variables from the CHELSA climate dataset [74,75]. This database consists of rasters with a horizontal resolution of 30 arcseconds (ca. 1 km) and integrates 85 bioclimatic variables, 6 of which were selected according to the criteria of Guisan et al. [32], that is, with a Pearson correlation equal to or less than 0.7 and low collinearity, with a variance inflation factor (VIF) value of less than 10. To filter the variables in this way, the vifcor function of the R package ecospat [76] was used. The selected variables were as follows: isothermality (bio 03), annual range of air temperature (bio 07), precipitation seasonality (bio15), mean monthly precipitation amount of the coldest quarter (bio19), growing degree days heat sum above 10 °C (gdd10), and accumulated precipitation amount on growing season days modeled by TREELIM [77] (gsp); see Table S2 for details.

Ensemble forecasting, using methods explained in detail in [32,78,79], was performed using the R package biomod2 [79,80] considering 14 valid occurrences of *A. moorei* and three algorithms (Random Forest, Maxent, and GLM). The ratio used to split the original database for calibration and testing was 70/30. Five scenarios of random pseudo-absences were considered, with 15 repetitions per run for a total of 225 combinations. From these, only those whose evaluation threshold resulted in a True Skill Statistics (TSS) value greater than 0.8 were selected, and an ensemble was generated according to the mean weighted by the TSS value of each selected input model. 

A total of 10 CMIP6 future scenarios [81] were included from the CHELSA climate dataset [74], considering five Global Circulation Models (GCMs: GFDL-ESM4, UKESM1-0-LL, MPI-ESM1-2-HR, IPSL-CM6A-LR, and MRI-ESM2-0) as proposed by [82], with two contrasting RCPs (SSP1-RCP2.6 and SSP5-RCP8.5). The scenario considered the time range of years 2041–2070, hereafter 2055.

### 2.4. Vulnerability to Climate Change

We generated a Climate Change Vulnerability Index (CCVI) for each *A. moorei* population according to the following procedure: (i) For each future scenario, we compared the present and future model to determine the degree of change in each cell of the distribution of the species. The objective was to determine how distant each population is from its minimal conditions for survival in the future. The first step consisted of overlaying the present scenario with the future scenario to obtain the part of the present distribution that corresponds to the future distribution. Then, (ii), the set of the habitat suitability values for the future distribution were extracted. These values were scaled using the “scale” function of the terra R package [83]. We used the center value to characterize the minimum value of suitability for the population to survive (i.e., the minimum suitable habitat as output by the ensemble forecasting modeling). This value represents the limit between vulnerable and not vulnerable. Finally, (iii), each of the scaled grid values was multiplied by (−1) to obtain a vulnerability gradient where ≤0 indicates no vulnerability to climate change, while positive values indicate increasingly high levels of vulnerability. In other words, under this index, populations within the range of suitable climatic variables that are therefore not vulnerable to climate change, reach zero or negative values in the grid cell. To the contrary, positive CCVI values indicate that climatic conditions are not suitable for the species. Consequently, the higher the CCVI value, the greater the vulnerability of a population to climate change. (iv) The resulting CCVI values in the grid were then extracted for all *A. moorei* individuals in each population using the extract function of the raster R package [84]. (v) The mean CCVI was then calculated for each population and each scenario.

We used the CCVI value for each climate scenario, with the aim of searching for possible differences related to the population structure.

### 2.5. Relationships between Population Structure and Vulnerability to Climate Change

Based on the parameters used to characterize *A. moorei* at the population level, we evaluated possible relationships between those parameters and the corresponding mean CCVI values. For this purpose, linear models with the lm function of the stats R package [85] were used for continuous numerical values, while binomial models with the glm function of R were used for values corresponding to proportions, as recommended by [86]. At the level of all individuals of *A. moorei*, and to assess a possible change in stem length in reproductive plants in relation to vulnerability to climate change, a robust linear model between stem length and CCVI value was performed using the lmrob function of the robustbase R package [87]. The latter is a linear regression that is not sensitive to outliers, as recommended for this type of data [88].

## 3. Results

### 3.1. Demographic Differences among Populations

The total number of individuals recorded for the species was 1615. The number of plants per population ranged from 4 to 298; see Table 1. The thirteen populations covered an elevation range from 637 to 1143 m a.s.l. The total area of occupation of the species fails to exceed 5 ha. The plant density ranged from 79 to 2383 plants per ha, with an average density for the species of 792 plants per ha. The mean distance between plants varied between 4 and 127 m in each population, with a mean of 40 m for the species. Young plant density varied from 16 to 863 plants per ha, with a mean of 234 plants per ha. The original census data are provided in Table S3. 

**Figure 3:**
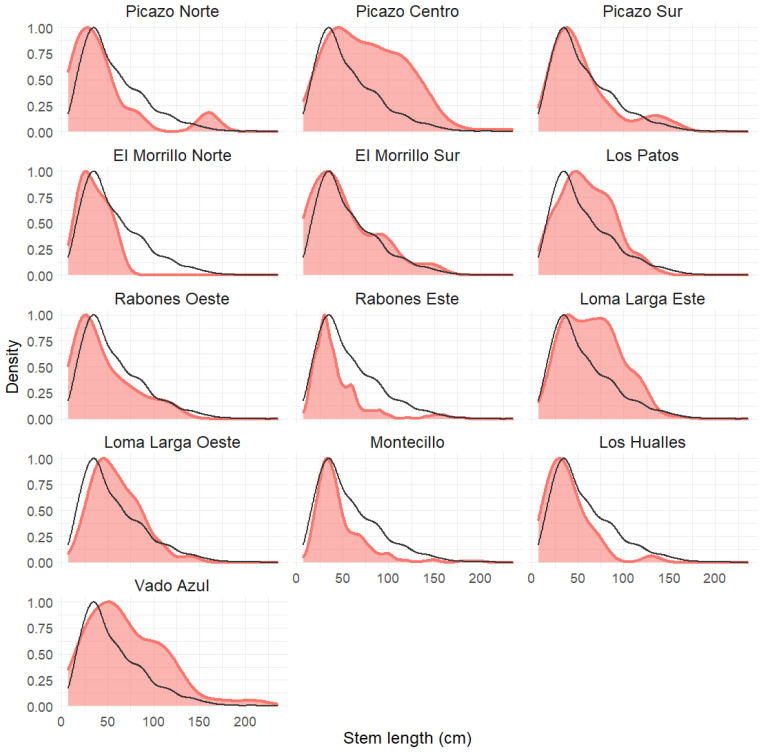
The size structure for each *A. moorei* population as per a scaled kernel density. The black line shows the size structure for the species as a whole for comparison with each population.

The population structure of each population according to its scaled kernel density is shown in Figure 3. The colored curve shows the stem size structure, while the black line shows the same for the species as a whole. Table 1 shows the number of plants and mean stem length for each population. The skewness values were positive and significant in all cases, indicating that there is currently a recruitment of young plants (with shorter stems) in all populations, as shown in Table 1. Likewise, Figure 3 shows that there is variation among populations in terms of the proportion of long stem plants (older plants), with high proportions in some populations (e.g., Picazo Centro, Los Patos, Loma Larga Este, and Vado Azul), and low proportions in other populations (e.g., El Morrillo Norte, Rabones Este, Montecillo, and Los Hualles). All the populations except El Morrillo Norte showed fruiting plants. 

### 3.2. Vulnerability to Climate Change in Relation to Population Structure

**Figure 4:**
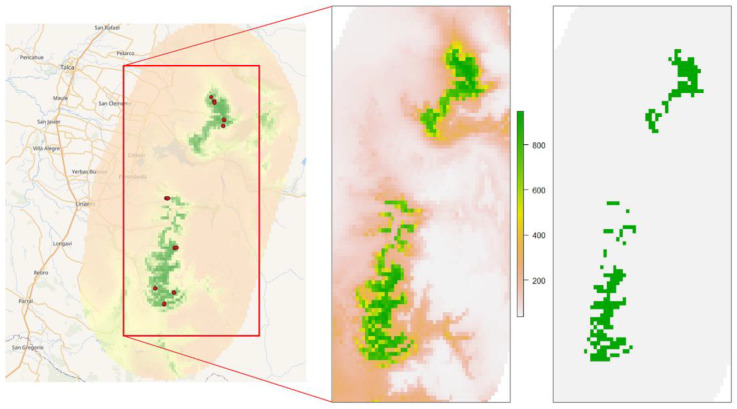
Ensemble forecasting model for *A. moorei* (present scenario). The left image is the total ensemble model, with all known populations as red dots. The middle image shows the gradient in the habitat suitability index. The right image is the binary model considering the biomod2 threshold of minimum suitable habitat.

On the 30 arcseconds grid (ca. 1 km) that we used, the 1615 precisely located individuals of *A. moorei*, grouped into 9 populations, contributing to 14 valid occurrences for SDM modeling. The ensemble model for the current distribution of *A. moorei* yielded a TSS value of 0.978. The gradient of the modeled distribution and its limit given by the threshold provided by biomod2 (value 747 out of a maximum of 1000) are shown in Figure 4.

Future projections according to the ensemble model under the different climate change scenarios indicated that none of the current locations of *A. moorei* populations will remain suitable for their climatic niche for the time scale of the study. The CCVI values corresponding to all populations indicate high vulnerability to climate change for this species in general; see Table 1. The CCVI values for each plant by climate scenario are shown in Table S3.

### 3.3. Relationships between Population Parameters and Vulnerability to Climate Change

**Figure 5:**
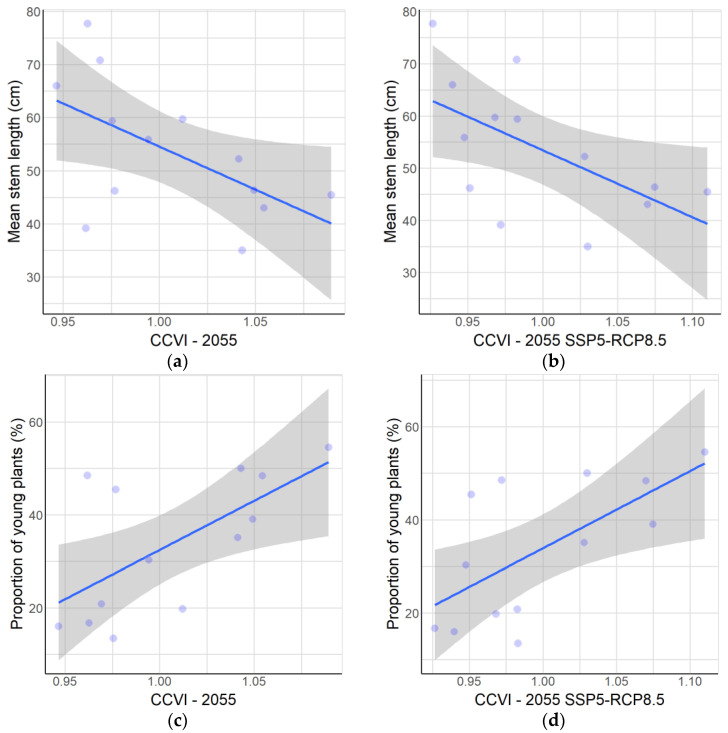

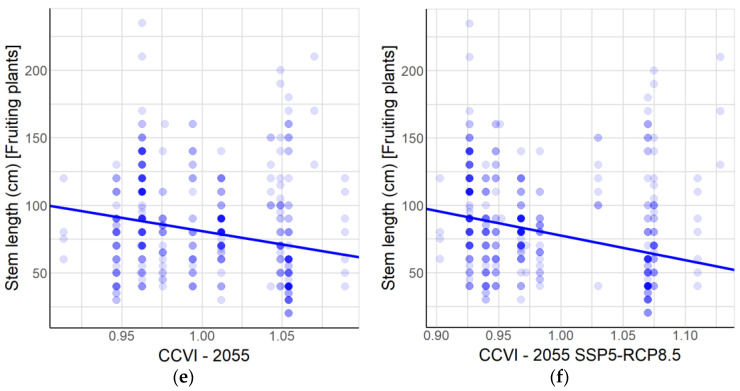
Relationships between population parameters and vulnerability to climate change. At the population level: (**a**) Mean stem length compared to CCVI at year 2055 as average. (**b**) Mean stem length compared to CCVI for 2055 SSP5-RCP8.5 scenario. (**c**) Proportion of young plants compared to CCVI at year 2055 as average. (**d**) Proportion of young plants compared to CCVI for 2055 SSP5-RCP8.5 scenario. At the level of all individuals of *A. moorei*: (**e**) Stem length of fruiting plants compared to CCVI at year 2055 as average. (**f**) Stem length of fruiting plants compared to CCVI for 2055 SSP5-RCP8.5 scenario. The statistical parameters are given in Table 2.

Figure 5 shows the statistically significant models that account for the relationships between the population parameters of *A. moorei* and the values of the climate change vulnerability index. The results of these models are presented in Table 2.

At the population level, an inverse relationship was found between the mean stem length and CCVI, both for the average of all climate change scenarios evaluated (*p*-value = 0.040; see Figure 5a) and for the average of the scenarios corresponding to the SSP5-RCP8.5 scenario (*p*-value = 0.035; see Figure 5b). We found a direct relationship (*p*-value < 0.001) between the proportion of young plants and the CCVI value for the average of all scenarios (Figure 5c) and also for the average corresponding to the SSP5-RCP8.5 scenario (*p*-value < 0.001, Figure 5d).

At the level of individual plants, there was an inverse relationship (*p*-value < 0.001) between the height of fruiting plants and the CCVI value, both for the average of the climate change scenarios analyzed (Figure 5e) and for the average scenario corresponding to SSP5-RCP8.5 (Figure 5f).

## 4. Discussion

In this study, we extended the known distribution of *A. moorei* from a latitudinal range of 10 km [67,70] to 71 km in the western foothills of the southern Andes. However, the area where its populations grow is still very small, with a total area of less than 0.05 km^2^. Therefore, its classification as an endangered species is unlikely to change. It is worth noting that populations of this species appear to be naturally fragmented within their native forest habitat. There are many areas close to the known populations that maintain their native forest habitat yet lack *A. moorei*. It would be worthwhile to continue the search for *A. moorei* guided by the distribution model developed in this study.

The field sampling effort detected 1615 individuals, allowing the use of 14 valid occurrences for the SDM. It is important to note that several studies have addressed the effect of the number of valid occurrences on the quality of SDMs. They agree that although a higher number of occurrences is always desirable for better accuracy, models for species with a narrow environmental range or rare species with limited environmental tolerances can be highly stable and reliable even when generated from a very small number of occurrences. However, there is disagreement on the minimum number of valid occurrences for appropriate use in SDMs, with minimum numbers of five [89], ten [90,91,92], fourteen [93], or eighteen [94]. We considered fourteen valid cells to be sufficient for the SDM of *A. moorei*, considering that it has a very restricted distribution with a narrow climatic niche. 

We showed that, independently of population, plants add on leaves at a rate of around 0.6 leaves per cm of stem, with this rate remaining fairly constant throughout the life of the plant. We took advantage of this finding and were able to establish a direct relationship between plant size and age. Because of this relationship, stem length in *A. moorei* became useful for understanding the demography of the species. We were able to verify that the seeds can regenerate in the natural environment (Figure S3). A preliminary germination test in the nursery (Figure S4) showed that 70% of the seeds were able to germinate. 

The demographic structure varied among populations of *A. moorei*. In all populations, there was the recruitment of young plants, as reflected in the skewness values (Table 1) and in the density peaks reached in smaller size classes (Figure 3), corresponding to young plants. On the other hand, the contributions of the medium and large size classes, corresponding to adult plants, showed much variation. Significantly, we found an inverse relationship between stem length (or adult age) and vulnerability to climate change (in both CCVI 2055 and CCVI SSP5-RCP8.5 scenarios). Since plant size is a good predictor of plant performance in terms of growth, fecundity, and survival [65], the lower representation of certain adult size classes in some populations could be understood as a trend toward earlier mortality in populations that are more vulnerable to climate change. 

There is much debate about the potential effects of ongoing climate change on plant longevity. Some authors suggest an increase in population persistence due to increased longevity [95,96], while others have observed that adverse environmental conditions can affect the survival of large plants by severely reducing their lifespan or longevity [47]. In an epiphyte transplantation experiment, a reduction in longevity was observed as a result of reduced water availability in the face of climate change [97]. Our results appear to be consistent with [47] and [97], as we found a relationship between reduced longevity in populations that are predicted by SDMs to be more vulnerable to climate change.

We detected a direct relationship between the proportion of young plants and vulnerability to climate change for both the mean CCVI scenario and the mean CCVI SSP5-RCP8.5. It may seem contradictory to find relatively more young plants in populations facing greater vulnerability to climate change. However, when we studied the relationships between CCVI and the parameters evaluated at the level of individuals, we discovered that the length of the stem in fruiting plants decreases as CCVI increases, which suggests that, in the face of greater vulnerability to climate change, plants initiate their reproductive stage at a younger age. The literature shows that climate change is altering environmental patterns and has been shown to potentially delay or even enhance regeneration by seeds [23]. Our results suggest that the greater vulnerability to climate change in *A. moorei* has provoked adaptive changes, whereby reproduction occurs earlier in plants with reduced overall longevity, thereby allowing them to cope better with rapid climate warming [10,98].

Of course, it could be asked whether variation in longevity in populations of *A. moorei* as seen today existed before climate warming became detectable in central Chile. Interestingly, the CCVI values extracted for the present climate scenario showed no relationship to the population parameter studied (the linear model corresponding to mean stem length versus CCVI showed a *p*-value = 0.413, while the binomial model corresponding to the proportion of young plants showed a *p*-value = 0.689), supporting the idea that trends have been established as of the time of climate change set in and are not related to the pre-climate change history of the species. On the other hand, at the individual level, a significant relationship was found between the stem length of fruiting plants and CCVI for the current climate scenario (*p* < 0.001), but it should be noted that this model has a slope of −13.6, which is significantly lower than the slope for each climate change model (CCVI 2055 and CCVI 2055 SSP5-RCP8.5 with slopes −196.7 and −182.9, respectively), as derived from the confidence intervals calculated by the confint function of the stats R package [85]; see Table S4. The latter could indicate that the demographic differences did exist before climate change set in but have become magnified recently. If this is the case, the onset of reproduction is expected to be even earlier in the future. 

The census conducted on *A. moorei* showed that all populations are currently recruiting young individuals. Therefore, this species has the potential to persist for some years to come. However, the long-term projections of SDMs paint a highly unfavorable situation for the persistence of our species. Populations of *A. moorei* were shown to vary greatly in size. Notably, some, but not all of the very small populations, had a high CCVI (e.g., El Morrillo Norte, Rabones Oeste, El Morrillo Norte, and El Morrillo Sur). The above results call for ex situ conservation efforts such as the rescue of propagules or germplasm that would favor the conservation of the species in the long term, placing special emphasis on the most vulnerable and especially the smaller, highly vulnerable populations. At the same time, there is a need to explore areas that could enable assisted migration, thereby helping the species to establish itself in similar forest communities and areas whose niche range of climatic variables is expected to remain favorable in the face of future climate change scenarios. We observed that some plants died in some of the populations with a high CCVI index (e.g., Rabones Este, Figure S5), suggesting unfavorable local microsite conditions. Such conditions should be the subject of further research. We did not find a clear pattern in the trajectory of populations with respect to their elevation: CCVI values are not related to elevation for the scenarios with a mean CCVI (*p* = 0.265) or a mean CCVI SSP5-RCP8.5 (*p* = 0.094). This is not surprising given the limited elevational range of *A. moorei.* However, upward elevational migration could constitute another means of adverting the adverse effects of climate change.

The data collected during the census of *A. moorei* populations have the potential to serve as a basis for exploring the future demographic pathway of each population. It would be interesting to calculate birth and death rates in a population viability assessment approach [99,100,101,102] so as to analyze the trajectory of each population in terms of stability, decline, or increase. We expect those populations that are shown to have plants with shorter lifespans to continue in that direction.

In summary, we interpret our results in *A. moorei* as evidence of a shift toward reduced plant longevity under greater vulnerability to climate change, as inferred from the future projections of the SDMs. To our knowledge, the relationship between changes in plant population structure and projected climate change effects, as inferred from SDMs, has not been explicitly addressed, nor has it been suggested in the many reviews on plant strategies to address climate change [1,4,5,11,15,103,104]. Our results show that an endangered plant species, despite its restricted distribution, appears to be rapidly adapting its local population structure to changes in climate, at least for the time being. 

Finally, the novel Climate Change Vulnerability Index (CCVI) proposed here allowed us to examine the differences in vulnerability to climate change between populations of *A. moorei*. Clearly, however, the use of this index is limited to models that are primarily based on climate variables or that depend on climate change in future scenarios. The CCVI can also be used to compare the vulnerability of different species to climate change since it is a standardized value. Other proposed indices with similar objectives use the difference between raw values (e.g., [43]), which prevents comparisons between different species, or they are designed for an entire species, which allows comparisons between different species but not between different populations within each species [105].

## 5. Conclusions

This study extends the distribution of endangered *A. moorei* from 6 to 13 populations and increases its latitudinal range from 10 to 71 km. Our finding suggests that many highly endemic species in the central Chilean hotspot are likely to have wider distributions than currently known.

A novel Climate Change Vulnerability Index (CCVI) was developed, which allowed an examination of the differences in vulnerability to climate change at the population level in *A. moorei*. For this endangered species, we found a significant inverse relationship between the stem length (or adult plant age) and vulnerability to climate change at the population and individual levels, and a direct relationship between the proportion of young plants and vulnerability to climate change. These results suggest a trend toward reduced plant longevity in populations that are most vulnerable to climate change. Overall, our results suggest that climate change in *A. moorei* is provoking adaptive change whereby plant longevity is reduced and reproduction occurs earlier. This endangered plant species, despite its restricted distribution, appears to be adapting its local population structure to changes in climate.

## Figures and Tables

**Table 1:** Population parameters for each *A. moorei* site, area, and CCVI.

Population *	Elevation (m a.s.l.)	Plants (n)	Mean Stem Length (cm)	Fruiting Plants (n)	Young Plants (n)	Adult Plants (n)	Skewness	Mean Distance between Plants (m)
Picazo Norte	951	11	46.2 ± 42.7	1	5	6	1.88	27.3 ± 26.1
Picazo Centro	919	209	77.7 ± 42.0	103	35	174	0.56	22.2 ± 13.5
Picazo Sur	889	109	55.9 ± 35.5	33	33	76	1.32	9.5 ± 5.3
El Morrillo Norte	979	4	35.0 ± 15.8	0	2	2	0.37	4.1 ± 1.7
El Morrillo Sur	895	77	52.2 ± 35.9	10	27	50	0.97	36.4 ± 23.6
Los Patos	1086	298	59.7 ± 28.7	71	59	239	0.36	21.8 ± 13.2
Rabones Oeste	795	22	45.5 ± 30.9	7	12	10	1.14	16.7 ± 9.6
Rabones Este	907	283	43.0 ± 28.8	78	137	146	2.39	92.0 ± 86.8
Loma Larga Este	1143	181	66.0 ± 30.8	49	29	152	0.38	53.1 ± 44.9
Loma Larga Oeste	1057	89	59.4 ± 26.3	26	12	77	0.79	11.7 ± 7.1
Montecillo	637	251	46.4 ± 28.4	35	98	153	2.41	50.6 ± 36.0
Los Hualles	750	33	39.2 ± 24.5	2	16	17	1.67	45.9 ± 39.6
Vado Azul	772	48	70.8 ± 41.7	7	10	38	1.02	126.7 ± 104.3
	**Area (ha)**	**Plant density (n/ha)**	**Young plants density (n/ha)**	**Adult plants** **density (n/ha)**	**Mean CCVI**		**Mean CCVI SSP1-RCP2.6**	**Mean CCVI SSP5-RCP8.5**
Picazo Norte	0.044	251.1	114.2	137.0	0.977 ± 0.081		1.003 ± 0.068	0.951 ± 0.086
Picazo Centro	0.159	1318.3	220.8	1097.5	0.963 ± 0.086		0.999 ± 0.065	0.927 ± 0.090
Picazo Sur	0.046	2383.1	721.5	1661.6	0.994 ± 0.083		1.041 ± 0.050	0.948 ± 0.083
El Morrillo Norte	0.002	1726.3	863.1	863.1	1.043 ± 0.066		1.056 ± 0.056	1.030 ± 0.075
El Morrillo Sur	0.480	160.6	56.3	104.3	1.041 ± 0.067		1.054 ± 0.056	1.028 ± 0.074
Los Patos	0.295	1010.2	200.0	810.2	1.012 ± 0.105		1.056 ± 0.070	0.968 ± 0.116
Rabones Oeste	0.055	402.0	219.3	182.7	1.089 ± 0.053		1.069 ± 0.056	1.110 ± 0.040
Rabones Este	0.977	289.8	140.3	149.5	1.054 ± 0.036		1.039 ± 0.028	1.070 ± 0.036
Loma Larga Este	0.618	293.0	46.9	246.1	0.947 ± 0.087		0.954 ± 0.076	0.940 ± 0.097
Loma Larga Oeste	0.046	1947.0	262.5	1684.4	0.976 ± 0.061		0.968 ± 0.057	0.983 ± 0.065
Montecillo	0.716	350.7	136.9	213.8	1.049 ± 0.052		1.024 ± 0.024	1.075 ± 0.060
Los Hualles	0.366	90.2	43.7	46.5	0.962 ± 0.082		0.952 ± 0.050	0.972 ± 0.104
Vado Azul	0.609	78.8	16.4	62.4	0.969 ± 0.133		0.956 ± 0.096	0.983 ± 0.161

* The populations are listed in latitudinal order.

**Table 2:** Statistical data from models of relationships between population parameters and climate change vulnerability.

Variable Y (Parameter)	Variable X (Scenario)	Type of Regression	Statistic	*p*-Value	R-Squared	Number of Data	Shapiro–Wilk’s W for Residuals	*p*-Value
Mean stem length (cm)	CCVI 2055	Linear model	F = 5.42	0.040	0.330	13	0.955	0.6822
Mean stem length (cm)	CCVI 2055 SSP5-RCP8.5	Linear model	F = 5.79	0.035	0.345	13	0.925	0.2893
Proportion of young plants (%)	CCVI 2055	Binomial model	Z = 9.15	< 0.001	0.670 *	13	0.964	0.8105
Proportion of young plants (%)	CCVI 2055 SSP5-RCP8.5	Binomial model	Z = 9.68	< 0.001	0.723 *	13	0.974	0.9396
Stem length (cm) (Fruiting plants)	CCVI 2055	Robust linear model	F = 23.14	< 0.001	0.057	422	-	-
Stem length (cm) (Fruiting plants)	CCVI 2055 SSP5-RCP8.5	Robust linear model	F = 44.69	< 0.001	0.105	422	-	-

* McFadden’s R-squared as an approximation for binomial models.

## Data Availability

The GPS coordinates of *A. moorei* plants are not made publicly available to avoid the collection of live plants *in situ* for ornamental use or trade. The data may be shared in justified cases upon request by the lead author. Vouchers from new sites have been deposited in the Universidad de Concepción herbarium (CONC).

## Supplementary Materials

The following supporting information can be downloaded at: https://www.mdpi.com/article/10.3390/plants12102017/s1, Figure S1: Image of an adult plant of *Anemone moorei* growing in its natural understory habitat; Figure S2: Growth pattern of the leaves on the stem of *A. moorei*; Figure S3: *A. moorei* seedlings from the Loma Larga Este population. Red arrows indicate pairs of green cotyledon leaves. Yellow arrow indicates the first true leaf with its distinct spiny margin; Figure S4: Germination of *A. moorei* in nursery. (a) Seeds prior to sowing. (b) A seedling with its cotyledons. (c) Seedlings with their first true leaves with spiny margins—the red arrows indicate remaining cotyledons; Figure S5: *A. moorei* plants that suddenly died in Rabones Oeste population; Table S1: Sampling of leaves per stem per year and number of leaves per 30 cm stem segment per population; Table S2: List of environmental variables selected to model the distribution of *A. moorei* according to Karger et al. [75]; Table S3: Census of stem lengths showing maturity stage in populations of *A. moorei*, and CCVI values; Table S4: Statistical data from models of relationships between population parameters and the index of climate change vulnerability applied to the present scenario in *A. moorei*.
