# Supplementary material for: Population-Based Evidence of Climate Change Adaptation in an Endangered Plant Endemic to a Biodiversity Hotspot
Source: Plants (Basel). 2023 May 18;12(10):2017. doi: 10.3390/plants12102017 (PMC10222059; doi:10.3390/plants12102017)
Supplement: Supplementary file 1 [file plants-12-02017-s001.zip › Alarcon_et_al_Plants202304_Supplementary_Materials_Figures.pdf]

Supplementary materials

Population-based Evidence of Climate Change Adaptation in an Endangered Plant Endemic to a Biodiversity Hotspot

Diego Alarcón [1,2]*, David Santos [3] and Mary T. K. Arroyo [1,2,4]

1 Departamento de Ciencias Ecológicas, Universidad de Chile, Santiago, Chile
2 Instituto de Ecología y Biodiversidad (IEB), Chile
3 Vivero Encanto Salvaje, Linares, Chile
4 Cape Horn International Center (CHIC), Chile
* Correspondence: chilebosque@gmail.com

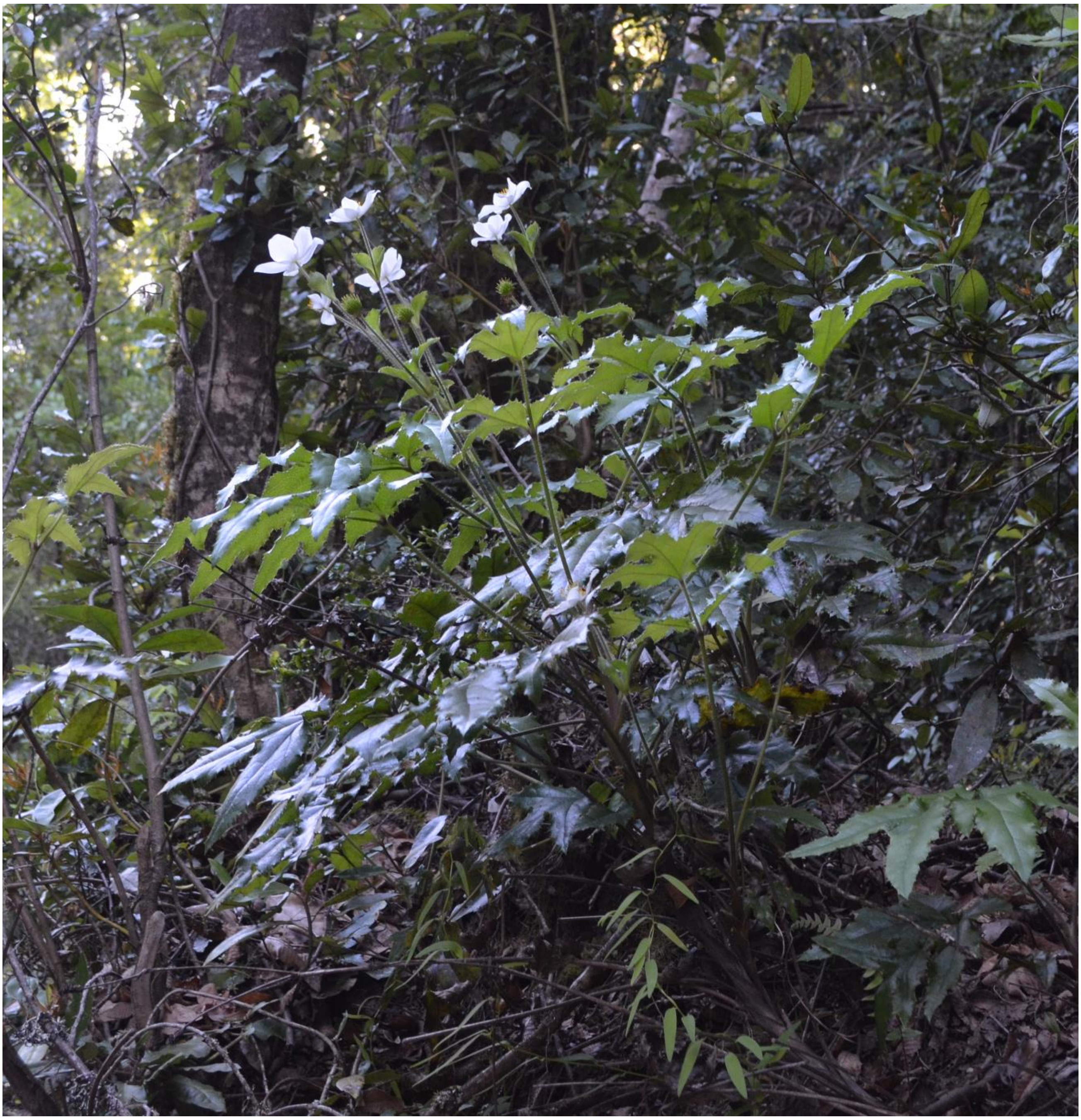

Figure S1: Image of an adult plant of *Anemone moorei* growing in its natural understory habitat.

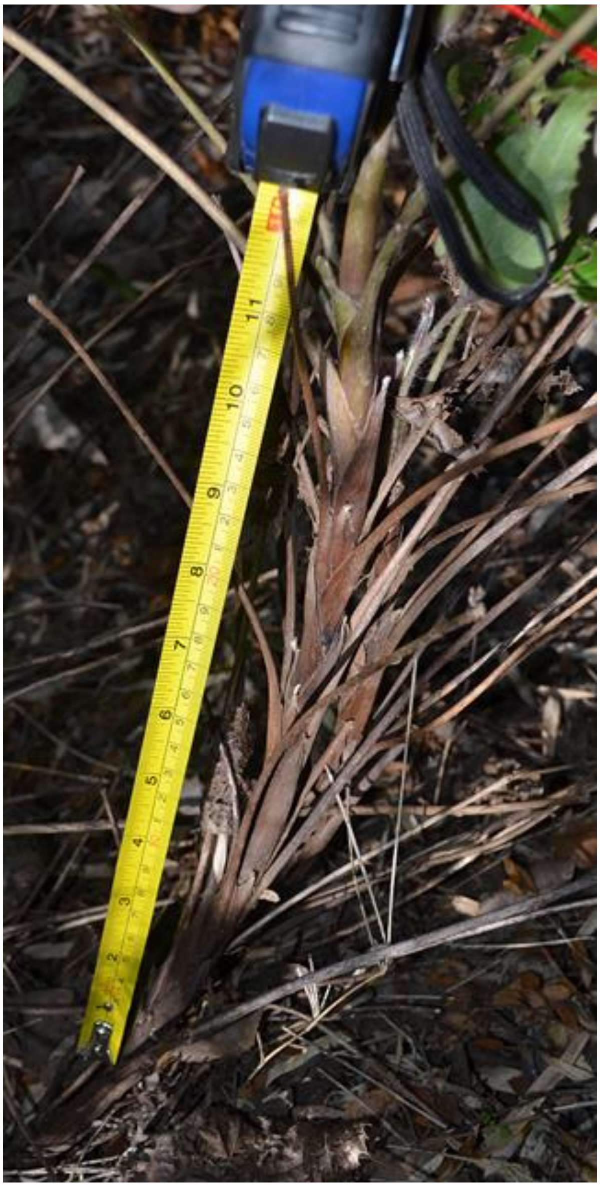

Figure S2: Growth pattern of the leaves on the stem of *A. moorei*.

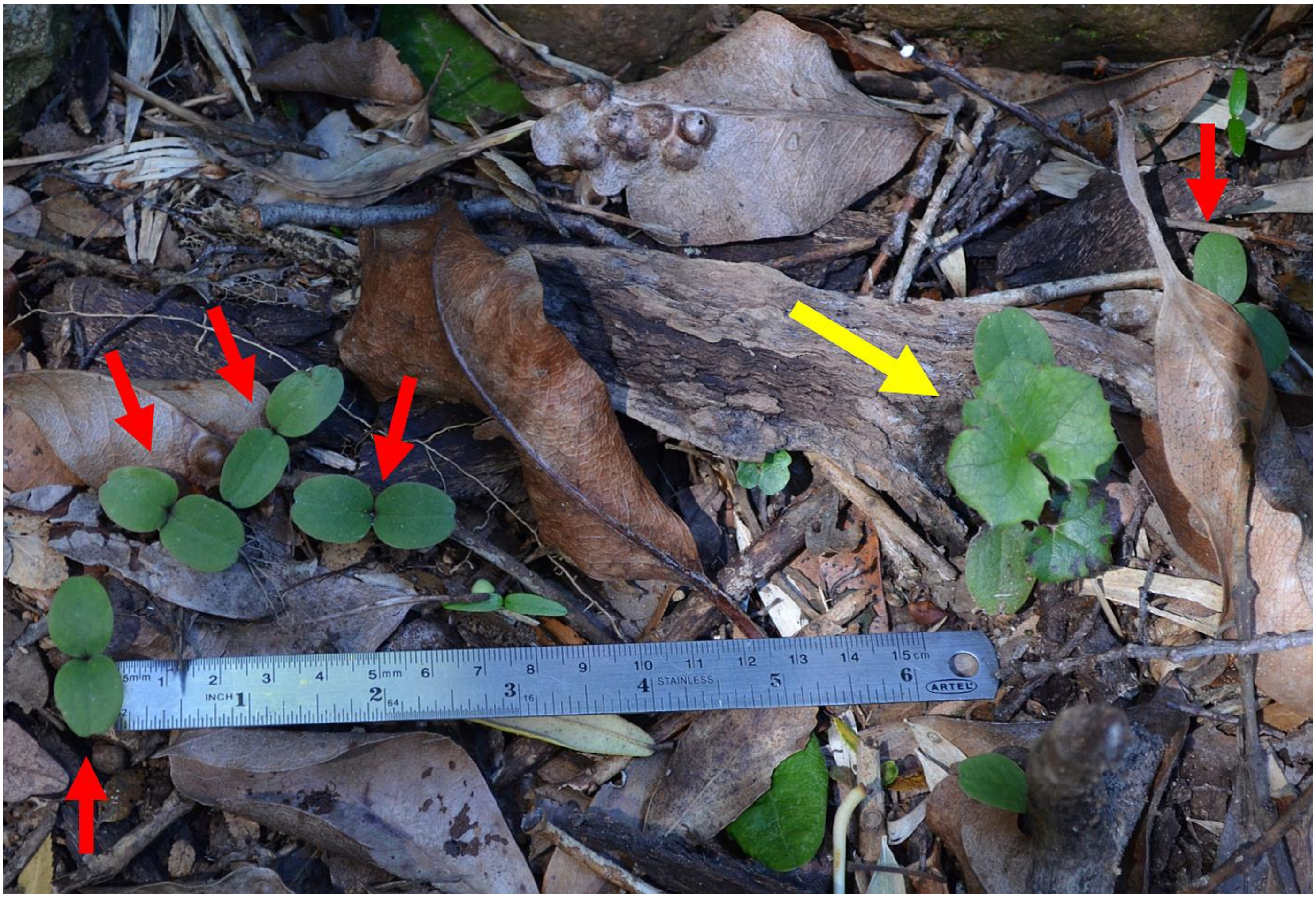

Figure S3: ***A. moorei*** **seedlings from the Loma Larga Este population. Red arrows indicate pairs of green cotyledon leaves. Yellow arrow indicates the first true leaf with its distinct spiny margin.**

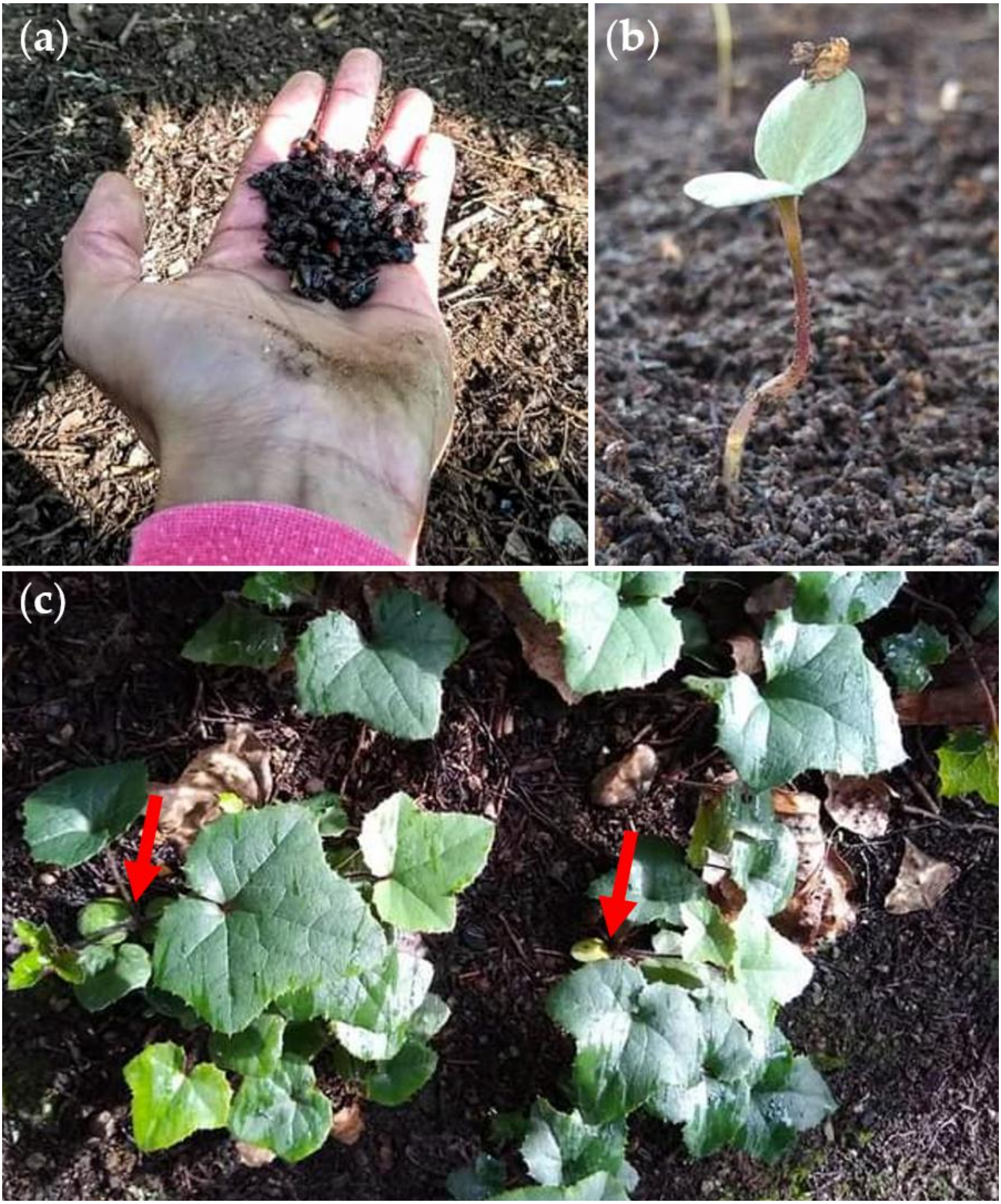

Figure S4: Germination of *A. moorei* in nursery. a) Seeds prior to sowing. b) A seedling with its cotyledons. c) Seedlings with their first true leaves with spiny margins, the red arrows indicate remaining cotyledons.

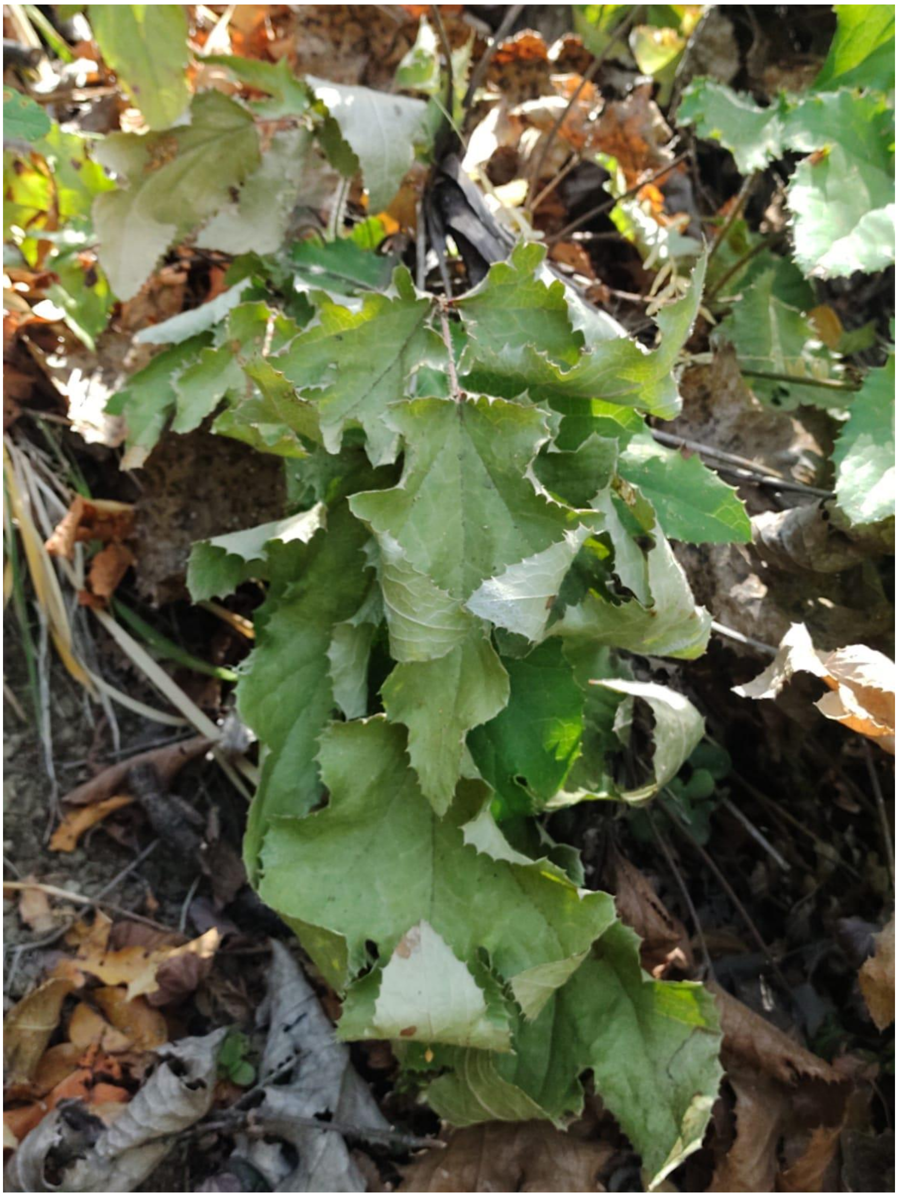

Figure S5: ***A. moorei*** **plants that suddenly died in Rabones Oeste population.**
